# Early onset Parkinson’s disease in the cycle of 3,4-methylenedioxymethamphetamine and substance use: a case report

**DOI:** 10.1186/s13256-023-04147-x

**Published:** 2023-09-23

**Authors:** Tianyi Hui, Song Guo

**Affiliations:** National Addictions Management Service, Institute of Mental Health, 10 Buangkok View, Buangkok Green, Medical Park, Singapore, 539747 Singapore

**Keywords:** Amphetamine, 3,4-Methylenedioxymethamphetamine (MDMA), Parkinson’s disease, Case report

## Abstract

**Background:**

Current evidence linking the development of Parkinson’s disease after the use of 3,4-methylenedioxymethamphetamine is mixed and limited, with only a few positive case reports demonstrating this.

**Case presentation:**

We examine this interesting case of a 49-year-old Chinese gentleman who used 3,4-methylenedioxymethamphetamine and subsequently developed early onset Parkinson’s disease at age 38 years. He had a family history of Parkinson’s disease, though the onset of his symptoms was significantly earlier than those of his family members. MDMA was a likely precipitating factor for the early onset of his symptoms. He then conversely used methamphetamines to augment his treatment of Parkinson’s symptoms. In the treatment of his Parkinson’s disease, dopamine replacement therapy and deep brain stimulation could perpetuate addictive behaviors such as dopamine dysregulation syndrome, and similarly perpetuate substance use in vulnerable individuals. He had also been diagnosed with a human immunodeficiency virus infection at age 43, and his antiretroviral therapy contributed to depressive symptoms, which then complicated the management of his substance use.

We examined the importance of managing his subsequent psychiatric and medical comorbidities to prevent their debilitating psychosocial impacts.

**Conclusions:**

This case implies that 3,4-methylenedioxymethamphetamine use may precipitate the early development of Parkinson’s disease in patients with genetic vulnerability. This highlights the risk in patients potentially paradoxically using substances to alleviate symptoms of Parkinson’s, which can in turn perpetuate the disease process.

## Background

The existing evidence linking substance use disorders to Parkinson’s disease is mixed. The neurochemistry of Amphetamine-type substances including 3,4-methylenedioxymethamphetamine (MDMA/Ecstasy) and Methamphetamine/Amphetamine works by acutely increasing synaptic dopamine. In the longer term this causes dopamine transporter changes and the structural degeneration of dopaminergic neurons in the substantia nigra [1], similar to those in patients with Parkinson’s disease [2]. Despite these suggestive pathological changes, clinically there are only isolated case reports of MDMA users developing Parkinsonian features[3–5]. Existing reports also do not examine the interplay of Parkinson’s disease and these patients’ subsequent substance use.

Early onset Parkinson’s disease is defined as onset before age 50 years. These patients have an earlier mortality, a longer duration of symptoms and thus meaningful years of life lost, and more complications from pharmacological treatment [6].

We examine this case of an individual who used MDMA and subsequently developed early onset Parkinson’s disease at age 38 years, on a background of a family history of Parkinson’s. He then conversely used Methamphetamines to augment his treatment of Parkinson’s disease.

## Case presentation

The patient in this case is referred to as Mr. X. He was a 49-year-old Chinese gentleman who was diagnosed with early onset Parkinson’s disease at age 38 years. He was single, living with his mother, and working as an ad hoc journalist. He had been referred to the Addictions Service in our hospital following a suicide attempt after his arrest for substance use. In Singapore, the Central Narcotics Bureau enforces penalties including imprisonment and heavy fines for the possession or consumption of controlled substances. Mr. X had been afraid of the legal consequences and overdosed on benzodiazepines as a result. He was unconscious with respiratory depression on his initial presentation to a medical hospital. In the intensive care unit (ICU), he was intubated with airway support. He recovered quickly and was extubated within a day. After medical stabilization he was transferred to the Addictions Service in our hospital for management of his substance use and mood. He was diagnosed with an Adjustment Disorder in reaction to his impending legal case.

Mr. X first started using MDMA at age 32 years during a trip to Hong Kong, where he had been introduced to the substance by a friend. On his return he continued to ingest three tablets of an indeterminate amount of MDMA daily. He would occasionally inject Ice (Methamphetamines) and snort Ketamine.

At age 38 years he presented with an asymmetrical stiffness and freezing in his gait, which led to multiple falls. He saw a neurologist and was diagnosed with early onset Parkinson’s disease. He had a positive family history of Parkinson’s disease. His mother’s maternal uncle, father’s eldest sister, and father’s paternal uncle’s son had the disease. Mr. X developed the disease at a significantly younger age than his family members, who were diagnosed in their 60s. He was started on medication: Ropinirole, a dopamine agonist, up to 2 mg three times a day, and Selegiline, a Monoamine Oxidase inhibitor, at 5 mg two times a day. After 2 years he was initiated on Levodopa and Benserazide at 125 mg daily for dopamine replacement, and later Entacapone 200 mg four times daily was added to prolong Levodopa’s action. Despite this, his symptoms progressed. He fell frequently. He had difficulty coping with his work due to bradykinesia, and could only take on part-time jobs as a journalist despite his holding a Bachelor’s degree in Arts. At age 47 years, he underwent bilateral Deep Brain Stimulation (DBS) electrode insertion into his subthalamic nuclei. This improved his episodes of stiffness. However, he continued to suffer from dyskinesias and bradykinesia.

As his Parkinson’s symptoms progressed, he began to use Ice predominantly to, in his words, “replace dopamine” to relieve stiffness between medication doses. He would inject the substance with an insulin needle. His neurologist titrated his medication according to his reported stiffness, but Mr. X would continue to use substances as he felt this was inadequate.

Mr. X was diagnosed with a Human Immunodeficiency Virus (HIV) infection at age 43 years. He had contracted the virus from a previous sexual partner. He had sexual intercourse since his teenage years, mostly with male partners and occasionally females. He would use Ice to enhance the sexual experience. He would also occasionally use Gamma-Hydroxybutyrate, benzodiazepines, and Amyl nitrite poppers.

After contracting HIV, he was initiated on an antiretroviral regimen of Tenofovir 300 mg, Lamivudine 300 mg, and Efavirenz 400 mg. His CD4 lymphocyte count remained well controlled, with the last count being 350 (reference range 288–1507). His viral load was not detectable.

He had no other past medical history.

His mood had been low for years following his diagnosis of Parkinson’s disease. He was frustrated by his falls and found his dyskinesias embarrassing. He did endorse feeling guilt and worthlessness, but no other biological symptoms of depression.

Mr. X was admitted to the ward under our Addiction Medicine Department and underwent a series of intensive counseling sessions two times a week with our Addictions Counselor. This was a cognitive behavioral therapy-based rehabilitation program involving group therapy, individual counseling, building community support, and relapse prevention planning. His family was involved in strategies to maintain support after he was discharged. His Parkinson’s medication timings were adjusted to suit his daily routine.

He was on Efavirenz for HIV treatment, which is known to cause neuropsychiatric side effects including depression. To mitigate the depressive effects of Efavirenz, his medication was changed to Abacavir 600 mg, Lamivudine 300 mg, and Rilpivirine 25 mg by his infectious disease specialist.

His mood improved and he was discharged from the ward. He was ambivalent about his cessation of drug use as he expressed his willingness to stop using drugs, however, he stated that using Ice would help him with his symptoms of Parkinson’s and depressed mood.

He was arranged to have follow-up appointments with our addictions doctors and counselors. However, he did not attend the appointments.

## Discussion

A review on the evidence of MDMA causing Parkinson’s disease showed mixed and limited evidence [7].

Amphetamine-type substances including 3,4-Methylenedioxymethamphetamine (MDMA/ecstasy) and Methamphetamine/Amphetamine acutely increase synaptic dopamine. This can later cause degeneration of dopaminergic neurons in the substantia nigra, which leads to the loss of dopamine in the basal ganglia [1]. Similar changes are found in patients with Parkinson’s disease [2].

There have been three isolated case reports of Parkinsonism developing in patients who used MDMA [3–5].

The first report published in 1999 described a 29-year-old man who developed a disturbance in his gait and fine coordination and had bradykinesia. He did not have a tremor. He had used MDMA on ten occasions in the prior year. Treatment with Levodopa and Pramipexole did not improve his symptoms[3].

The second report published in 2003 described a 19-year-old man who developed clinical symptoms of a resting tremor, decreased typing proficiency, and difficulty rising in the morning. He had used MDMA for 6 months prior. His symptoms were responsive to Trihexyphenidyl but not Selegiline [5].

The final report published in 2003 described a 38-year-old man who developed Parkinsonism that progressed to Hoehn and Yahr stage 5 within 4 years. He had used MDMA heavily almost 20 years ago, then a few times a year for 10 years. His symptoms responded to Ropinirole and Selegiline though his response deteriorated progressively. He underwent bilateral subthalamic Deep Brain Stimulation, which provided partial relief of his symptoms [4].

In contrast to the first two case reports, our patient’s Parkinson’s symptoms were responsive to Parkinson’s treatment. Our report also uniquely describes how our patient used substances to relieve symptoms of Parkinson’s, which has yet to be explored in current studies.

Conversely, a neuroimaging case–control study reported normal dopamine binding in MDMA users. They reported instead that serotonergic neurotoxicity of MDMA was more prominent [8]. MDMA has even been reported to have an anti-Parkinsonian effect in rats [9].

Mr. X had been using predominantly MDMA before his Parkinson’s symptoms, which presented 20 years before his family members’ did. This suggests the neurotoxicity of MDMA being a likely precipitating factor for Mr. X’s Parkinson’s disease.

Methamphetamine has been more clearly linked to the development of Parkinson’s disease. Methamphetamine users are twice as likely to develop the disease. A review examined the damage of dopaminergic fibers in the striatum and cell bodies in the substantia nigra, which proved to be similar to the degeneration seen in Parkinson’s disease [10]. Mr. X rarely used methamphetamine until after his Parkinson’s onset, making its contribution to the development of his disease less likely.

Early onset Parkinson’s disease has been defined as the age of onset being up to age 50 years. A positive family history is more common in this population of patients [6, 11]. Common mutations seen include the Parkin gene, which can result in a younger onset of symptoms [12]. These patients could also represent the younger presentations of Lewy body dementia [13].

Mr. X’s family history of Parkinson’s suggests an autosomal recessive pattern of inheritance. This pattern of inheritance is seen in the Parkin gene [14], though he did not have formal genetic testing done. However, Mr. X’s significantly earlier presentation of symptoms compared with his family suggests the contributory component of MDMA use.

HIV is known to result in cognitive and motor deficits similar to that in Parkinson’s [15]. In Mr. X’s case the onset of his infection 5 years after his Parkinson’s diagnosis and his undetectable viral load makes HIV a less likely contributory cause of his Parkinson’s.

Impulse control disorders (ICDs) are a known complication of dopamine agonist medications used in the treatment of Parkinson’s disease [16]. Mr. X’s treatment included dopamine agonists and medication to inhibit dopamine metabolism. Deep Brain Stimulation also leads to a higher risk of developing postoperative ICDs [17]. Though substance use in itself is not an impulse control disorder, impulsivity is a component in addictive disorders and it is prudent to consider the effects of medication in perpetuating addictive behaviors.

In the use of dopamine replacement therapy in the treatment of Parkinson’s, some patients develop an excessive desire for and use the medication beyond what is necessary to control their symptoms. This is known as Dopamine Dysregulation Syndrome, which can result in dyskinesias, mood dysregulation, anxiety, and impulse control disorders [18]. Though Mr. X did not abuse his Parkinson’s medication, he used Methamphetamine in a similar way to relieve his symptoms of stiffness. His Methamphetamine use complicated the titration of his medication, which ultimately resulted in him requiring Deep Brain Stimulation. This highlights the importance of monitoring the control of Parkinson’s symptoms to avoid the abuse of both medication and substances by patients, especially in those with known substance use disorders.

Depression is a common neuropsychiatric complication in Parkinson’s disease. Clinically significant depressive symptoms can occur in up to 35% of patients with Parkinson’s [19]. Through the disease process, degenerative changes in the noradrenergic and serotonergic neurons can cause mood disturbances [20]. In the treatment of Parkinson’s, dopamine agonists and Deep Brain Stimulation can cause mood changes including depression [21, 22].

Being cognizant of the neuropsychiatric effects of Parkinson’s and its treatment is especially important in Mr. X’s case, in which depressive symptoms are a common comorbidity in both substance use and HIV infections. The treatment of HIV itself through medications, including Efavirenz, can increase the risk of developing a depressed mood [23]. Mr. X’s multifactorial contributors for depression—Parkinson’s disease, substance use, and HIV treatment—made treating his depression challenging and continued to perpetuate his substance use.

## Conclusions

Amphetamine-type substance use can lead to the development of Parkinson’s disease. This can lead to debilitating psychosocial impacts, especially in the younger population of substance users. This case implies that MDMA use may precipitate the early development of the disease in patients with genetic vulnerability. The potential of patients to paradoxically use substances to alleviate symptoms of Parkinson’s should be closely monitored given the risk of developing addictive behaviors in Parkinson’s disease, both from the disease process and treatment. Managing psychiatric and medical comorbidities is important in optimizing treatment. Constant communication between subspecialty doctors and a multidisciplinary approach would be useful in allowing for early intervention and slowing the progression of disability.

## Data Availability

Data used in the study are available form the corresponding author on reasonable request.

## Abbreviations

DBS: Deep brain stimulation
ICDs: Impulse control disorders
HIV: Human immunodeficiency virus
MDMA: 3,4-Methylenedioxymethamphetamine
